# Increased Lipocalin-2 in the retinal pigment epithelium of *Cryba1* cKO mice is associated with a chronic inflammatory response

**DOI:** 10.1111/acel.12274

**Published:** 2014-09-25

**Authors:** Mallika Valapala, Malia Edwards, Stacey Hose, Rhonda Grebe, Imran A Bhutto, Marisol Cano, Thorsten Berger, Tak W Mak, Eric Wawrousek, James T Handa, Gerard A Lutty, J Samuel Zigler, Debasish Sinha

**Affiliations:** 1 Wilmer Eye Institute, The Johns Hopkins University School of MedicineBaltimore, MD, USA; 2 The Campbell Family Institute for Breast Cancer Research and Ontario Cancer Institute, University Health NetworkToronto, ON, Canada; 3 National Eye Institute, National Institutes of HealthBethesda, MD, USA

**Keywords:** age-related macular degeneration, *Cryba1* cKO mice, inflammation, lipocalin-2, lysosomes, retinal pigment epithelium

## Abstract

Although chronic inflammation is believed to contribute to the pathology of age-related macular degeneration (AMD), knowledge regarding the events that elicit the change from para-inflammation to chronic inflammation in the pathogenesis of AMD is lacking. We propose here that lipocalin-2 (LCN2), a mammalian innate immunity protein that is trafficked to the lysosomes, may contribute to this process. It accumulates significantly with age in retinal pigment epithelial (RPE) cells of *Cryba1* conditional knockout (cKO) mice, but not in control mice. We have recently shown that these mice, which lack βA3/A1-crystallin specifically in RPE, have defective lysosomal clearance. The age-related increase in LCN2 in the cKO mice is accompanied by increases in chemokine (C-C motif) ligand 2 (CCL2), reactive gliosis, and immune cell infiltration. LCN2 may contribute to induction of a chronic inflammatory response in this mouse model with AMD-like pathology.

AMD is one of the leading causes of blindness in developed countries. Several studies support the hypothesis that the immune system is involved in the pathogenesis of AMD, in concert with, or in addition to, other factors (Ambati *et al*., 2013; Whitcup *et al*., 2013). One factor that has captured our interest is βA3/A1-crystallin, which has been reported to be present in human drusen (Crabb *et al*., 2002), a precursor to AMD. We have recently shown in the Nuc1 rat (a spontaneous mutation in the *Cryba1* gene) and in mice lacking βA3/A1-crystallin (cKO) specifically in the RPE (Sinha *et al*., 2008; Valapala *et al*., 2014), impaired lysosomal clearance and decreased autophagy and phagocytosis. This leads to RPE cell degeneration, subretinal lesions in the posterior pole, and deposits between the RPE and Bruch's membrane, all characteristic changes in AMD (Zigler *et al*., 2011; Kaarniranta *et al*., 2013). We now report further degenerative changes in the RPE of aging cKO mice. Transmission electron microscopy (TEM) of the RPE from 12-month-old mice revealed RPE degeneration, including loss of basal infoldings, prominent intracellular vacuoles, undigested melanosomes and photoreceptor outer segments, and widening of adherens junctions in cKO mice compared to age-matched *Cryba1*^fl/fl^ controls (Fig.1A–D). We also observed a significant increase in cell death in the cKO RPE compared to controls (Fig.1E).

**Figure 1:**
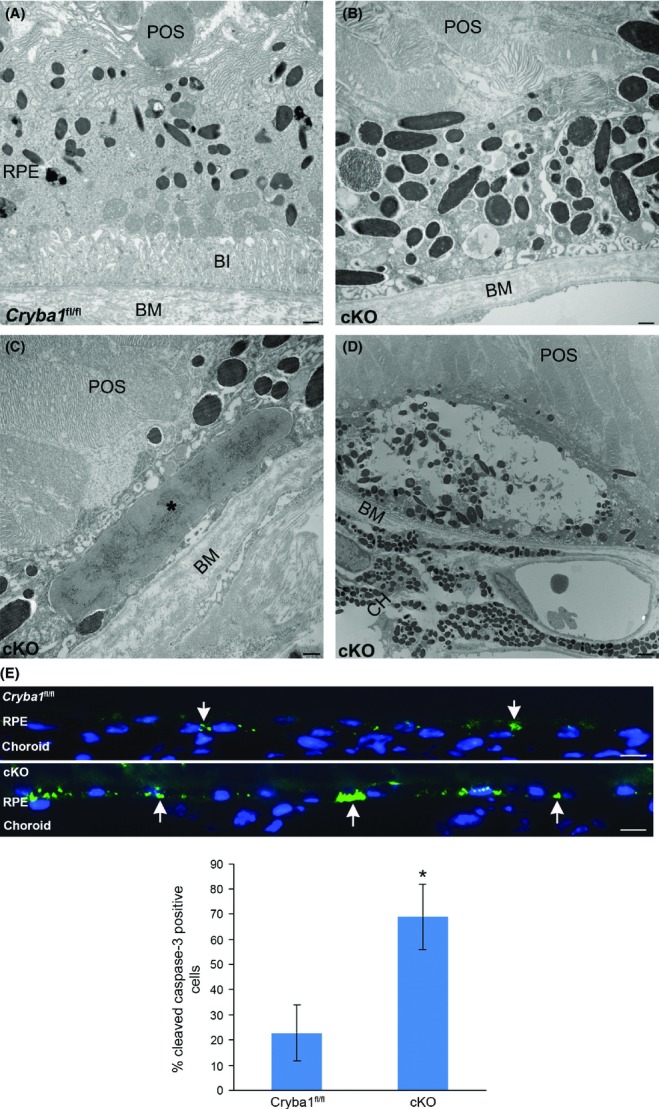
Degenerative changes in aging cKO retina: TEM (panels A–D) shows loss and truncation of basal infoldings (BI) and accumulation of undigested materials in 12-month-old cKO RPE (panel B), as compared to *Cryba1 *^fl/fl^ (panel A). Additional degenerative changes in cKO RPE include large intracellular deposits (* in panel C) and large vacuoles (panel D). Bar = 500 nm. E shows immunostaining of 12-month-old *Cryba1 *^fl/fl^ and cKO retinal sections with anticleaved caspase-3 antibody. A significant increase in cleaved caspase-3 positive cells (green, arrows) is seen in the cKO RPE compared to control. Nuclei stained with DAPI (blue). The histogram shows the percentage of total cells that are caspase-3-positive. Bar = 10 μm. BI: basal infoldings; BM: Bruch's membrane; POS: photoreceptor outer segments; CH: choroid (*P ≤ 0.05).

A recent study reported that aberrant lysosomal function can elicit innate immune responses (Hasan *et al*., 2013). In various tissues, the downstream consequences of lysosomal dysfunction usually include inflammation, resulting in pathogenesis (Lee *et al*., 2010). It is likely that disturbances in the homeostasis of RPE cells, due to accumulation of undegraded intracellular material in the cKO mice, trigger a para-inflammatory response in an attempt to restore normal RPE function. Para-inflammation is intermediate between the basal homeostatic state and a chronic inflammatory response.

Dysregulated para-inflammation leading to a chronic inflammatory state has been proposed to play an important role in other diseases, including type 2 diabetes, cardiovascular disease as well as in age-related retinal diseases (Medzhitov, 2008; Xu *et al*., 2009). Adipokines are involved in the pathogenesis of obesity, diabetes, and hypertension. We wondered whether they might also be acting in concert with impaired autophagy and phagocytosis to convert the para-inflammatory response to a chronic inflammatory response in cKO mice. To test this hypothesis, we performed an adipokine array and found that LCN2 protein was elevated in RPE cells from cKO mice relative to Cryba1 ^fl/fl^. Western analysis confirmed this finding in RPE cells from aging mice, but found no difference in younger mice (Fig.2A). LCN2 has been shown to be markedly elevated in serum and tissues during inflammation (Flo *et al*., 2004). As, in macrophages (Halaas *et al*., 2010), LCN2 is initially endocytosed into early endosomes and subsequently sorted to lysosomes, one would expect it to accumulate in cKO RPE with age, as lysosomal-mediated clearance is impaired in these mice. Interestingly, the mRNA expression of LCN2 is also upregulated in the RPE from aging cKO mice (Fig. S1). We have postulated that βA3/A1-crystallin regulates the mechanistic target of rapamycin, complex 1 (mTORC1) signaling in the lysosomes of RPE cells (Valapala *et al*., 2014). It is now accepted that mTORC1 is also a key regulator of gene transcription (Laplante & Sabatini, 2013). Thus, LCN2-mediated induction of inflammatory stimuli in the cKO RPE could result from increased LCN2 transcription as well as reduced LCN2 degradation.

**Figure 2:**
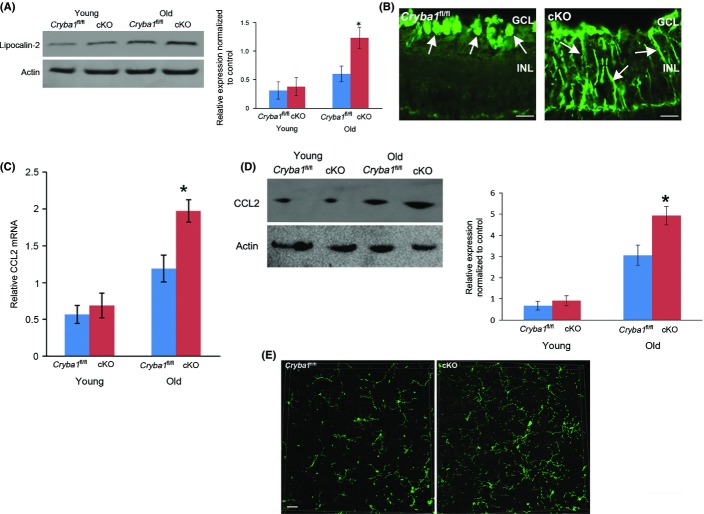
Evidence of LCN2-mediated inflammation in RPE of cKO mice: (A) Western analysis shows significant upregulation of lipocalin-2 protein in older cKO mice compared to age-matched controls. No significant change was found in younger animals. (B) In 12-month-old retina sections stained with GFAP antibody (green), staining is limited to the GCL in *Cryba1 *^fl/fl^ retina, characteristic of astrocytes (left panel, arrows). In the cKO retina (right panel), additional staining of Muller glia processes (arrows) indicates Muller cell activation. Bar = 10 μm. (C) qRT–PCR shows significant upregulation of CCL2 mRNA in older cKO mice (12 months) compared to the younger animals (4 month). (D) Consistent with the PCR results, protein levels of CCL2 were upregulated in older cKO mice compared to age-matched controls. (E) Retinal flat mounts stained with Iba1 antibody appear denser with extended processes in the cKO mice compared to *Cryba1*^fl/fl^. Bar = 30 μm (*P ≤ 0.05).

To confirm that an inflammatory response occurs in the aging retinas of cKO mice, we stained 12-month-old *Cryba1*^fl/fl^ and cKO retinal sections with an antibody against GFAP (glial-fibrillary acidic protein). GFAP staining of Müller cell processes indicates the cells are activated (Gehlbach *et al*., 2006), a condition associated with reactive gliosis and an inflammatory response. Our data revealed the presence of GFAP-expressing activated Müller cells in the retina of the cKO mice, but not in the *Cryba1*^fl/fl^ controls (Fig.2B). In the control retina, GFAP staining was only observed in the ganglion cell layer (Fig.2B), where astrocytes, for which GFAP is used as a marker, reside.

One of the molecules known to be produced during inflammation is the chemokine CCL2, which is upregulated in AMD (Raoul *et al*., 2010; Suzuki *et al*., 2012). Our results show a significant induction of CCL2 mRNA in the 12-month-old cKO mice compared to controls (Fig.2C). In contrast, at 4 months of age, levels of CCL2 mRNA were similar in *Cryba1*^fl/fl^ and cKO samples. These data are further supported by Western analysis of protein extracts from RPE/choroid preparations. The CCL2 protein levels were significantly higher (,65% increase) in the older cKO animals compared to controls, while in younger animals, they were not significantly different (Fig.2D). Interestingly, in LCN2 KO mice (Berger *et al*., 2006), GFAP levels in the RPE are similar to control, while CCL2 levels are lower than control (Figs S2 and S3), indicating that loss of LCN2 does not induce inflammation. The retinas of LCN2 KO mice appear to be anatomically normal (data not shown).

Tissue homeostasis is monitored by resident tissue macrophages that remain in a basal homeostatic state. Para-inflammation acts to maintain tissue homeostasis and to restore tissue function (Medzhitov, 2008). However, when tissue malfunction is excessive, additional macrophages are recruited, inducing chronic inflammation. Our data show that Iba1^+^ microglial cells, while present in both *Cryba1*^fl/fl^ and cKO retinas (Fig.2E), appear denser in cKO samples with extended processes. Interestingly, the infiltration of microglia into the cKO retina does not appear to be as dense as might be expected from a chronic inflammatory response. However, this is likely misleading due to the highly dynamic nature of the cellular interactions in the abnormal tissue and the fact that LCN2 has diverse functions and is known to participate in the self-regulatory elimination of activated microglia *in vivo* (Lee *et al*., 2007).

A variety of animal models have been developed over the years that simulate different aspects of AMD (Pennesi *et al*., 2012). Our mouse model exhibits a slowly progressing form of AMD-like pathology associated with inefficient lysosomal clearance. This could switch the para-inflammatory response to a chronic inflammatory immune response as the mice age. In summary, this study provides intriguing findings relevant to AMD, suggesting a linkage of LCN2 with chronic inflammatory responses.

## Supporting Information

**Data S1** Experimental methods. **Fig. S1** Quantitative reverse transcriptase (qRT) PCR using Taqman expression probes shows that lipocalin-2 message is significantly increased in the old cKO mice compared to age matched floxed controls (**P* ≤ 0.05). **Fig. S2** Left panel shows western blot for GFAP in RPE from wild type and LCN2-KO mice at 2 months and 9 months of age. **Fig. S3** Left panel shows western blot for CCL2 in RPE from 2 month and 9 month old wild type and LCN2-KO mice.
