# Supplementary material for: Increased Lipocalin-2 in the retinal pigment epithelium of Cryba1 cKO mice is associated with a chronic inflammatory response
Source: Aging Cell. 2014 Sep 25;13(6):1091–4. doi: 10.1111/acel.12274 (PMC4244249; doi:10.1111/acel.12274)
Supplement: Supplementary file 1 [file acel0013-1091-sd1.docx]

**EXPERIMENTAL METHODS**:

***Conditional knockout mice***

βA3/A1-crystallin conditional knockout (*Cryba1* cKO) mice were generated as described previously (Valapala *et al*., 2014). The controls used in this study are *Cryba1* ^fl/fl^ mice as the RPE phenotype between these mice and heterozygote *Cryba1* floxed/*Best1*-Cre-positive mice were similar. All animal studies were conducted in accordance with the Guide for the Care and Use of Animals (National Academy Press) and were approved by the Animal Care and Use Committee of Johns Hopkins University.

***Electron microscopy***

Samples were prepared and transmission electron microscopy was performed as described previously (Zigler *et al*., 2011).

***Antibodies***

The following antibodies were used in this study: LCN2 (AB2267, EMD Millipore, Billerica, MA, USA), CCL2 (506003, BioLegend, San Diego, CA, USA), GFAP (Z0334, Dako, Carpinteria, CA, USA), Iba1 (Wako Chemicals, Richmond, VA, USA), Cleaved caspase-3 (Cell Signaling Technology, Danvers, MA, USA).

***Immunofluorescence***

Immunofluorescence was performed as described previously (Zigler *et al*., 2011).

***Mouse Adipokine array***

The adipokine profile was obtained using a Mouse Adipokine Array Kit Proteome Profiler™ (R&D Systems, Minneapolis, MN, USA), following the manufacturers’ guidelines. The signal produced by the antibody reaction was detected with a Image Quant LAS 4000 (GE Healthcare Life Sciences, Piscataway, NJ, USA).

***SDS-PAGE and western blot analysis***

SDS-PAGE and western blot analysis were performed as described previously (Zigler *et al*., 2011).

***RNA isolation, CDNA synthesis and qRT-PCR***

RNA isolation, cDNA synthesis and qRT-PCR were performed as described previously (Zigler *et al*., 2011).

***Cell death assay***

The extent of cell death was determined by immunostaining 12 month old *Cryba1*^fl/fl^ and *Cryba1* cKO retinal sections with anti-cleaved caspase-3 antibody. Cell death was determined by counting the total number of cells and the number of caspase-3 positive cells in a minimum of 4 fields per section and an average of 4 sections were analyzed per group. Data are presented as percent of total cells that are caspase-3 positive.

***Statistical Analysis***

Statistical analysis was performed using Microsoft excel and GraphPad 5.0 software. The *P*-values were determined by two-tail Student’s *t*-test in a triplicate experiment representative of at least three independent experiments. Multiple comparisons were made using Kruskal-Wallis test. Significance was defined as *P≤0.05. Results are presented as mean ± SD. Each biological replicate has at least 3 technical replicates.

**Figure S1**: Quantitative reverse transcriptase (qRT) PCR using Taqman expression probes shows that lipocalin-2 message is significantly increased in the old cKO mice compared to age matched floxed controls (*p≤ 0.05).

**Figure S2**: Left panel shows western blot for GFAP in RPE from wild type and LCN2-KO mice at 2 months and 9 months of age. As shown in the right panel, no significant difference was found among the 4 samples when normalized to the actin control.

**Figure S3**: Left panel shows western blot for CCL2 in RPE from 2 month and 9 month old wild type and LCN2-KO mice. Right panel shows relative protein levels normalized to actin control. CCL2 was significantly lower in the LCN2-KO samples relative to the wild type samples at both ages. (*p≤0.05)
